# Assessing Livestock Production Practices on Small-Scale Multi-Species Farms Located on Floreana Island, Galápagos Islands

**DOI:** 10.3390/ani13040686

**Published:** 2023-02-16

**Authors:** Sarah Rhea, Blanca E. Camacho, Carrisa W. Amoriello, Maria Correa, Gregory A. Lewbart, Marilyn Cruz, Alberto Vélez, Paulina Castillo, Monique Pairis-Garcia

**Affiliations:** 1 College of Veterinary Medicine, North Carolina State University, Raleigh, NC 27607, USA; 2 Facultad de Veterinaria, Universidad de la República, Montevideo 13000, Uruguay; 3 Galapagos Science Center, Universidad San Francisco de Quito (USFQ) & The University of North Carolina (UNC) at Chapel Hill, Puerto Baquerizo Moreno 200150, Ecuador; 4 Agencia de Regulación y Control de la Bioseguridad y Cuarentena para Galápagos, Puerto Ayora 200350, Ecuador

**Keywords:** Galápagos Islands, animal welfare, production animal, antimicrobial resistance, ecosystem health, conservation

## Abstract

**Simple Summary:**

In the Galápagos Islands, food animal agriculture is an important source of local meat and eggs and is driven by smallholder farms. With anticipated future increases in production animal populations in the Galápagos—one of the most biodiverse and protected ecosystems on the planet—the health of humans, animals, and the environment must be considered. An integral component of sustainable agriculture is animal welfare, which considers the health, nutrition, housing, and behavior of animals. To our knowledge, no formal assessments of production animal welfare have been conducted in the Galápagos Islands. We evaluated animal welfare conditions on farms located on Floreana Island, Galápagos utilizing five basic measures of animal care. We identified opportunities for enhanced activities in two of these measures, animal health management and timely euthanasia. Future work should promote knowledge transfer and in-country capacity building in these areas. Efforts to positively impact smallholder farm livelihoods in the Galápagos Islands will sustainably support the interconnected realms of animal health and welfare, wildlife and environmental health, and food safety and security in this unique ecosystem.

**Abstract:**

Globally to date, established international standards for animal welfare, a priority of sustainable agriculture, have primarily focused on large-scale producers. However, across Latin America, including in Ecuador’s Galápagos Islands, smallholder farms play a critical role in food safety and security. We assessed five basic animal welfare measures (feed and water access, shelter availability and housing systems, animal health management, animal behavior, and timely euthanasia) for poultry, pigs, and cattle on Floreana Island, Galápagos. Utilizing assessment standards from multiple US sources and international standards, we developed a questionnaire and used it to conduct in-depth interviews during 4–5 July 2022 with eight participating producers, representing 75% of animal agriculture on Floreana. While we identified opportunities to enhance competencies in animal health management and timely euthanasia, farms performed well in the other assessed measures. Future work should promote knowledge transfer and in-country capacity building in farm biosecurity, access to veterinary care, antimicrobial resistance surveillance, and euthanasia methods. Efforts to positively impact smallholder farm livelihoods in Galápagos—one of the most biodiverse and protected ecosystems on the planet—will sustainably support human health through the interconnected realms of animal health and welfare, wildlife and environmental health, and food safety and security.

## 1. Introduction

The Galápagos Islands, a 21-island archipelago off the Ecuadorian coast, are one of the most biodiverse and protected ecosystems on the planet. Approximately 25,000 permanent residents live on the four human-inhabited Galápagos Islands, and over 270,000 tourist visits occurred in 2019 [1,2]. With the growth of travel, trade, and tourism in recent decades, anthropomorphic pressures are mounting and include growing demands for food production [3].

Food animal agriculture, although a relatively small industry, serves an important role in producing local meat and eggs for Galápagos residents [4]. An estimated 932 tons of meat was produced and consumed locally on the Islands in 2014; however, recent estimates suggest that 75% of the Island’s agricultural food supply are imported from mainland Ecuador [5]. Galápagean farm systems are typically smallholder mixed-production systems, including cultivated crops (sugar cane, yucca, corn) in conjunction with food animals such as poultry (layers and meat birds), pigs, and cattle. The Galápagos rely heavily on importation given the relatively small agricultural footprint of each island. This is driven by several factors including, restricted zoning areas for agriculture, limited access to sustainable technologies, species biodiversity, and negative historical perspectives on the role agriculture has played in conservation efforts [4]. Agricultural practices on the islands are subject to strict restrictions on imported products for production, including limitations on vaccine access and regulated imports on live chicks. In contrast to such restrictions, antimicrobial products for use in people or animals can be obtained without a prescription.

Over the past decade, concerted efforts have been underway to re-evaluate agricultural production in Galápagos. For example, in 2014 the Ecuadorian Ministry of Agriculture and Livestock developed a bio-agricultural plan for Galápagos with the intent to reduce reliance on imported products and boost local agricultural production [4]. Within this realm, research, technological developments, and innovation were prioritized as key strategies to improve the self-sufficiency and sustainability of Galápagean agricultural systems. With these initiatives, production animal populations in Galápagos will likely increase.

The anticipated expansion of food animal agriculture systems in Galápagos must ensure that the health of humans, animals, and ecosystems are safeguarded in a sustainable way [6,7]. At the interface of wildlife and environmental health and human health, livestock play an integral role in the emergence and transmission of zoonotic diseases [8]. For example, non-human hosts and the environment may serve as large and important reservoirs for pathogenic and antimicrobial-resistant bacterial strains and antimicrobial resistance genes [9,10]. In the Galápagos, antimicrobial resistance has been documented in finches and giant turtles close to agricultural areas and on beaches near human sewage discharge [11,12,13].

Animal welfare, which considers the health, nutrition, housing, and behavior of animals, is a priority for sustainable agriculture and directly relates to the United Nations Sustainable Development Goals [14,15,16]. By ensuring that basic animal welfare standards (e.g., access to clean water and appropriate nutrition, good management practices, and no overstocking) are met, animals can survive and thrive in current and future conditions and human and ecosystem health can be sustained [8].

Floreana Island, which at 17,125 hectares (ha) is the smallest of the four human-inhabited Galápagos Islands, has about 140 residents who rely on farming, tourism, and fishing for their livelihoods [17,18]. The Galápagos National Park manages more than 98% of Floreana’s land and the remaining 2% consists of an agricultural zone (290 ha) in the central highlands and the village of Puerto Velasco Ibarra (42 ha) [18]. Home to more than 50 species found on the International Union for Conservation of Nature Red List, including the Galápagos petrel (*Pterodroma phaeopygia*), Floreana mockingbird (*Mimus trifasciatu*), Floreana giant tortoise (*Chelonoidis elephantopus*), and medium tree finch (*Camarhynchus pauper*), Floreana and its diverse native species have been under threat from of invasive species, including rodents, for many years [17,18,19,20]. Restoration efforts on Floreana are underway which require the confinement of farm animal species to avoid rodenticide poisoning during implementation periods of invasive rodent eradication [21,22,23].

Animal welfare is typically assessed using tools that objectively evaluate and document existing animal welfare conditions and identify areas for future improvement [24]. On a global scale, assessments are implemented in both commercial and niche market systems to verify that minimal animal welfare standards are being met and motivate producers to continually advance animal welfare on farms. To date, the focus of animal welfare efforts in Latin America has been on large producers and at the industry level [25,26,27]. To our knowledge, no formal assessments of production animal welfare have been conducted in the Galápagos Islands. However, with the anticipated expansion of food agriculture systems and continued conservation efforts, understanding the status of animal welfare on farms in Galápagos is critical [6,7].

We initiated a research project on Floreana Island in the summer of 2022 to characterize production animal health, welfare, and management, including the use of antimicrobials on farms, and conduct baseline surveillance for antimicrobial-resistant *Escherichia coli* on farms. Here, we present one objective of the larger project. The objective of this study was to evaluate current animal welfare conditions on farms located on the island of Floreana utilizing five basic measures of animal care adapted from existing animal welfare assessment tools for poultry, pigs, and cattle.

## 2. Materials and Methods

We developed a questionnaire to conduct in-depth interviews with participating producers and guide the systematic collection of on-farm observations at each farm on Floreana Island (Supplementary Materials). The questionnaire was adapted utilizing assessment standards from multiple US sources (e.g., Beef Quality Assurance, National Chicken Council Welfare Guidelines, Certified Humane and Animal Welfare Approved), as well as international guidelines developed by the World Organisation for Animal Health [25,28,29,30,31,32]. These standards were selected based on the authors’ experience utilizing such standards in previous on-farm assessments. To accurately assess animal welfare baseline for farms located on Floreana Island, basic animal welfare measures were selected to serve as minimum standards for animal welfare required for animals to survive. The selected measures did not include more advanced approaches to assessing welfare that focus on positive affective states and the animal’s ability to thrive.

The questionnaire was composed of two sections: farm characteristics (e.g., number and type of animals, number of workers) and basic animal welfare measures (feed and water access, shelter availability and housing systems, animal health management, animal behavior, and timely euthanasia). The questionnaire, which was developed in English and translated to Spanish, contained multiple choice and semi-closed questions.

**Figure 1:**
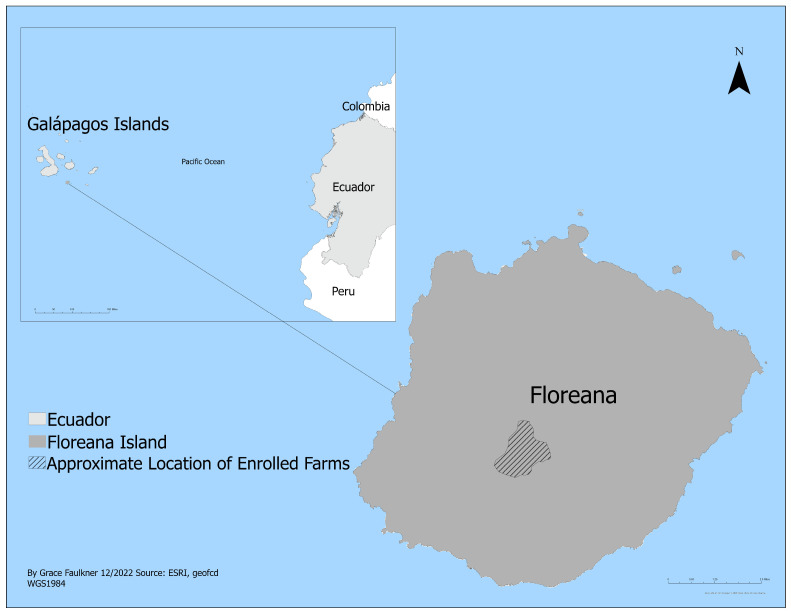
Floreana Island map with general area of enrolled farms indicated.

During 4–5 July 2022, we conducted scheduled site visits to eight participating multi-species farms on Floreana Island. These eight farms, located in the central highlands area, represent approximately 75% of total livestock production on Floreana Island (Figure 1). Each farm visit lasted approximately 2 h during which a face-to-face interview with the producer was conducted in Spanish by a lead interviewer (BEC) using the questionnaire, with assistance from members of the study team (CWA, MC, and MPG). Animal behavior was evaluated by one of the authors (MPG) for all farms. This individual has over 10 years of experience assessing animal livestock behavior and is a board-certified diplomate in the American College of Animal Welfare. Over 15 stereotypic behaviors were evaluated categorized as either oral or locomotory for all species (for specific behaviors assessed, please refer to Mason and Rushen, 2006) [33]. Behaviors were identified as abnormal if they were different from the population of animals on-farm or differed in pattern, frequency, or context for that species. This included but was not limited to destructive behavior (e.g., tail biting, feather pecking, cannibalism) and behaviors performed in increased quantity outside of the scope of the species behavioral repertoire (e.g., aggression, inactivity, unresponsiveness to stimuli). All data were collected on paper and subsequently transferred into Epi Info^TM^ (Centers for Disease Control and Prevention, Atlanta, GA, USA) for data management and analysis.

The results and conclusions presented are based on verbal information provided by the interviewed producers and on-farm evaluations made by the study team. Descriptive statistics were conducted in Epi Info^TM^ and Microsoft Excel (Redmond, WA, USA). This work was approved by North Carolina State University’s (NCSU) Institutional Review Board (protocol #20629) and was considered exempt from NCSU’s Institutional Animal Care and Use Committee, as animals were only observed. Informed consent was obtained from all study participants.

## 3. Results

### 3.1. Farm Characteristics

The eight participating farms ranged from 1 ha to 110 ha in size. All eight farms had poultry, and seven had two or more species, including pigs and cattle (Table 1). Farm work was most consistently performed by two or three individuals, including the farm owner(s). For farms with these species, the mean number of production animals on the farms were as follows: 90 (median: 65) poultry (eight farms), 26 (median: 14) swine (six farms), and 28 (median: 24) cattle (five farms). The study team observed wild birds and the presence of rodents at each of the eight farms. Six (75%) farms had one or more dogs, five (62.5%) farms had one or more cats, and four (50%) farms had one or more equines on the property. Across the enrolled farms, we observed a lack of quarantine areas to introduce new animals and limited or damaged fencing and penning. All eight farms had at least one footbath, typically at the entrance to a concrete animal enclosure, though not all were in use during the site visit.

### 3.2. Basic Animal Welfare Measures

#### 3.2.1. Feed and Water Access

All eight enrolled farms collected rainwater for production animal consumption and two farms (25%) used geomembranes to enhance water harvesting and storage. In the Floreana context, geomembranes are well-structured ponds, lined and covered by plastic materials, to help facilitate the collection and storage of rainwater and watershed run-off. Most commonly, however, the water used for animals was rainwater collected in tanks or cisterns; other sources included lagoons, water from the house, or water purchased in-town. A natural spring on Floreana was an additional resource noted by interviewed producers. Water sources were accessible to all animals and to the producers’ knowledge, free of contaminates. No animals demonstrated clinical signs of dehydration, suggesting access to water sources was not limited.

All farms relied on cultivated crops as the primary food source for animals with the addition of commercial feed and supplements purchased within the previous year. Animal feed type varied and included foodstuffs such as yuca, corn, sugarcane, bananas, molasses, watermelons, and papaya, all grown on site. Cattle were typically also pastured. At the time of the interview, only two farms (25%) reported having at least a 20-day stockpile of animal feed available on-farm in case of emergency. At the time of the farm visit, no animal with poor body condition was identified, across all species. Producers acknowledged the use of body condition as a factor to help the decision-making process in respect to implementing timely euthanasia. Feed provided daily for all species maintained good health, met the physiological requirements for all species across all production stages, and supported natural foraging behavior for all species. Aggressive behavior at the feed access site was also not noted, suggesting feed quantity and availability were sufficient for the herd size.

#### 3.2.2. Shelter Availability and Housing Systems

**Figure 2:**
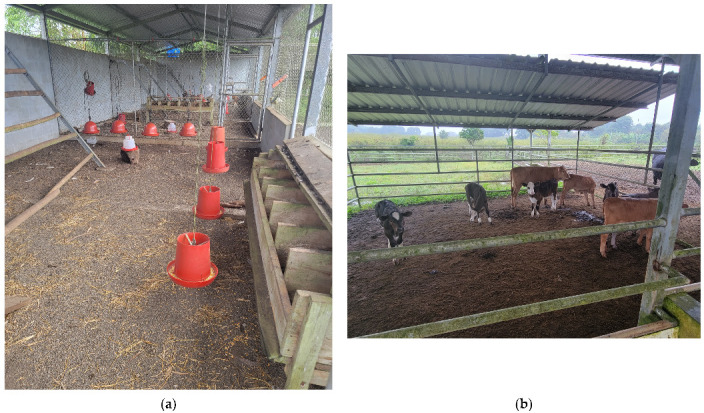
Images of shelter structures on enrolled farms taken by the study team with producer permission at the time of site visits (4–5 July 2022): (**a**) All enrolled farms had structures for poultry, as exemplified by this poultry house at one enrolled farm; (**b**) One farm had permanent shelter for cattle, and other farms with cattle were reportedly in the process of constructing similar shelter structures.

All eight enrolled farms had permanent shelter structures for poultry and swine, which typically consisted of concrete flooring with indoor/outdoor access (Figure 2a). Poultry shelters also included netting to prevent interaction between poultry and wild birds; however, at most farms this netting was incomplete or torn, preventing the complete physical separation of species. Wild birds were observed eating directly from poultry feeders at several farms. Shelters served to protect animals from extreme weather and provide a protected area for animals to be housed during the widespread distribution of rodenticide across the Island as part of the rodent eradication program [18]. Only one farm had a permanent shelter completed for cattle, which consisted of dirt flooring, gates, and metal roofs covering approximately one third of the pen (Figure 2b). Other farms with cattle were in the process of constructing similar shelter facilities. Although facility design varied across farms, no animal was physically restrained (e.g., tethered) and housing systems were designed to reduce disease transmission and minimize risk associated with injury. All farms had the ability to isolate individual animals if needed, and brief observations of animal handling on farms suggested producers had sufficient handling training and could utilize facilities to humanely handle and manage animals if needed.

#### 3.2.3. Animal Health Management

All farms reported parasitic infection as a common livestock condition observed on-farm (Table 2). Floreana Island had no permanent resident veterinarian. However, a 6-month rotating agricultural worker position that supported crop work and some veterinary technician activities was reportedly filled at the time of the site visits. Seven farms (87.5%) reported consulting with a governmental veterinarian by phone or having an in-person consultation with a governmental veterinary technician within the previous month. One farm (12.5%) reported previous monthly on-farm visits from a veterinary technician to provide guidance on a specific animal health challenge.

During the 12 months prior to the site visit, three farms (37.5%) reported using injectable penicillin; other antibiotics reportedly used at enrolled farms included oxytetracycline, sulfamethoxazole/trimethoprim, ceftiofur, and tylosin. Antibiotics were not consistently stored per manufacturer guidelines. Five farms (62.5%) reported using piperazine during the previous 12 months; other antiparasitics reportedly used at enrolled farms included levamisole and fenbendazole. Antibiotics and antiparasitics were typically administered under the guidance of a veterinarian or veterinary technician. Five farms (62.5%) reported basing their decision on when to stop antimicrobial (i.e., antibiotic or antiparasitic) administration on the advice of a veterinarian or a veterinary technician, and two (25%) reported basing their decision on the treatment time recommended by the manufacturer. None of the enrolled farms maintained animal health records on the farm; therefore, morbidity, mortality and culling, and reproductive rates could not be used as assessment criteria as suggested in the World Organization for Animal Health Terrestrial codes [25].

#### 3.2.4. Animal Behavior

No abnormal behaviors or stereotypies were present at the time of the assessment. However, seven farms (87.5%) described occasional feather pecking historically among chickens and increased aggression among swine. Incidences of these behaviors were noted more commonly during times of physical restriction.

#### 3.2.5. Timely Euthanasia

When asked about the five key indicators (e.g., behaviors, clinical signs) of suffering in livestock—unable to stand, poor body condition, unable to access feed and water, severe injury, and unresponsive to treatment (see Supplementary Materials)—none of the farms identified all five (Table 2). Two farms (25%) identified two to three of the five indicators. Six (75%) reported zero to one of these clinical signs as an indicator of suffering. Six (75%) reported that suffering animals on the farms are euthanized. The most common mode of euthanasia reported was exsanguination. Most farms lacked access to euthanasia tools such as captive bolt guns or electrocution.

## 4. Discussion

An animal’s health and its welfare are strongly correlated. Animals in states of disease have compromised welfare and frequently experience poor affective states (i.e., pain, suffering, distress) as a byproduct of disease [34]. To improve animal welfare from a health perspective, the control of current and future disease impacts must be prioritized. Farm systems with systemically healthy, well-cared-for animals (e.g., access to feed, water, and shelter) produce safe food while ensuring human and ecosystem health [8,15]. Globally, pastoral settings of smallholder farms are inherently less intensive, potentially allowing producers to provide more attention to individual animals. However, these producers are often faced with limited resources compared to larger systems. Challenges in animal welfare on these smallholder farms can originate from scarce feed and water, limited access to veterinary guidance, or an absence of knowledge [15].

When conducting animal welfare assessments, it is critical that the findings be interpreted based on performance criteria outcomes [25]. Numerous factors, including local customs, resource availability, and farm size, lead to heterogeneity across systems in different settings [35]. Additionally, unique initiatives, such as the conservation efforts and sustainable development planning in the Galápagos, should be considered when interpreting animal welfare assessment results [6]. Considering these complexities, issues raised in this assessment could be addressed using national agricultural extension approaches in partnership with national Universities [36].

We assessed five basic measures of animal care, namely feed and water access, shelter availability and housing systems, animal health management, animal behavior, and timely and humane euthanasia, on Floreana Island. For their animals, all eight enrolled farms provided shelter, water, and appropriate diets to maintain proper body condition. We found no evidence of abnormal behaviors or stereotypies among the animals, and producers reported experience in identifying and mitigating abnormal behaviors during past periods of animal confinement. We identified opportunities for technical assistance and education in two of the five assessed measures—animal health management and timely euthanasia.

Animal health management: Strategies to control animal disease vary and can be categorized as either prevention or management. Prevention strategies aim to eliminate disease introduction into farm systems and minimize disease spread if such introduction occurs. These prevention strategies include strict biosecurity measures (e.g., physical barriers, well-maintained footbaths) and pharmaceutical interventions (i.e., vaccinations) to facilitate herd immunity and minimize disease impact. Management strategies to control animal disease focus on the rapid detection of disease followed by aggressive and appropriate animal care to minimize disease impact at the individual and herd level. These management strategies include maintaining animal health records with treatment details and accessing veterinary consultation when needed.

The farms enrolled in this study had limited to no physical biosecurity protocols in place at the time of our site visits. Specifically, we noted a lack of designated quarantine areas for the introduction of new animals, damaged or incomplete fencing and penning, and unused footbaths. Given the limited number of inhabitants on the island and the physical location of the farms in the highlands at a distance from the town (Figure 1), non-designated human access onto farms may not be a significant biosecurity concern on Floreana. However, we did observe direct contact between wild birds and poultry. Wildlife and livestock can serve as pathogen reservoirs for one another, and direct or indirect contact of these reservoirs with susceptible populations can drive the development of emerging infections and facilitate disease spread [37].

Access to veterinary consultation for medical care and treatment, including antimicrobial administration, was available to the Floreana farms. However, due to the remote nature of Floreana and the fact that veterinary support on the Island was limited, most veterinary consultations were conducted via telephone calls to mainland Ecuador. Reportedly, on-farm in-person veterinary consultations occurred less frequently. None of the enrolled farms maintained animal health records.

Current policies in Ecuador permit access to and use of antibiotics and antiparasitics in farm animals without a veterinary prescription. Most enrolled farms reported treating sick animals based on clinical signs of disease and administering antibiotics and antiparasitics according to drug label instructions and, in some cases, under veterinary guidance. However, two farms reportedly stop administering antibiotics when they think the animal has improved enough or has not improved at all. We found that antimicrobial treatment protocols varied on and among farms, even for similar disease conditions. Currently, comprehensive data regarding antimicrobial use, antimicrobial residues, and antimicrobial resistance over time and across the farms of Floreana are not available.

In production systems such as Floreana Island where resources are limited (e.g., limited access to a veterinarian, vaccine import restrictions), herd health management is uniquely challenging. Given the physical isolation of the Island, alternative means to achieving routine access to veterinary care should be explored. For example, routine visits (e.g., quarterly or semi-annually) from mainland veterinary personnel to establish a veterinarian–client–patient relationship could be supplemented with more formalized telehealth services [38]. The effectiveness of telehealth services would be enhanced through the consistent use by farms of simple, standardized animal health records. Although the implementation of any telehealth services on Floreana must consider the Island’s limited broadband internet connectivity, approaches to overcome this challenge could be borrowed from strategies for human medicine telehealth in remote settings [39].

Evidence-based on-farm biosecurity protocols should be implemented, with an initial focus on high-impact and low-resource interventions. These interventions might include providing stricter physical barriers (i.e., restoring fencing and penning, repairing torn poultry house netting), designating a new animal quarantine area, and consistently using well-maintained footbaths. Ongoing efforts to enhance existing farm facility structures (e.g., cattle shelter facilities) should continue. Training opportunities that leverage established best-practices in lower resource settings and are developed specifically for the Galápagos Island producers could positively impact the implementation of enhanced biosecurity activities on farms.

Systematic monitoring of antimicrobial use and antimicrobial resistance can guide potential interventions to mitigate the spread of resistant organisms and resistance genes among livestock, wildlife and the environment, and humans [40]. In the Galápagos, these interventions could be at the farm, Island, or national level. Integrated antimicrobial resistance surveillance across animal, environmental, and human sectors using a One Health approach is the goal [41]. However, initial efforts in the Galápagos could prioritize active surveillance of antimicrobial use in animals and resistance indicator organism(s) of epidemiological importance. For example, extended-spectrum beta-lactamase producing *Escherichia coli* are considered a preferred indicator by the World Health Organization for integrated human, animal, and environmental antimicrobial resistant surveillance, particularly in lower resource settings where resistance baselines have not yet been established [42]. Such efforts could be synchronized with other animal disease or zoonotic disease surveillance activities. Citizen science approaches to antimicrobial resistance surveillance data collection could be considered, engaging producers in these efforts, and ensuring that surveillance summaries are provided back to producers and other local stakeholders. Training opportunities specific to the Galápagean context could include information about judicial use and appropriate administration of antimicrobials. The presented study is part of a larger research project on Floreana Island that included collecting livestock fecal samples during the farm visits to determine the on-farm presence of extended-spectrum beta-lactamase producing *Escherichia coli*. A separate research report outlining our microbiological methods and results from that portion of the larger research project is forthcoming.

Timely Euthanasia: Euthanasia is a critical tool used by producers and veterinarians to end the suffering of a compromised animal (e.g., an animal that fails to respond to treatment or is catastrophically injured). To ensure good welfare is being met, the experiences and affective states of animals from birth to death must be considered. Euthanasia techniques vary by species, but all euthanasia methods should render an animal immediately unconscious followed by cardiac and respiratory arrest. Euthanasia techniques should also consider handling and restraint time and should prioritize techniques that minimize or eliminate any additional pain and distress to the animal.

Based on our interviews with producers in the study, on Floreana Island cattle are euthanized primarily with gunshot, while poultry and pigs are euthanized via exsanguination while conscious. Two farms reported not practicing euthanasia at all, even if they have sick or compromised animals. Although gunshot is an approved method for euthanasia for cattle, per the American Veterinary Medical Association, exsanguination alone in poultry and pigs is not, as animals are conscious throughout the process [43]. Work conducted on halal slaughter in poultry suggests birds that are exsanguinated without stunning demonstrated a three-fold increase in cortisol compared to baseline levels collected in lairage [44]. Similarly, Gregory and colleagues reported in their 2008 study that birds exsanguinated while sensible may experience prolonged periods of pain and distress as brain function is maintained for a longer period in non-stunned birds [45]. Previous studies dating back to 1977 suggest catecholamine response in pigs is elevated during the exsanguination process [46].

Given this previous work, and our ethical obligation to minimize pain and distress in animals, exsanguination should be eliminated as a stand-alone technique for euthanasia on Floreana Island. Instead, producers should consider alternative techniques that first render the animal completely unconscious prior to death. Such examples include cervical dislocation in poultry, blunt force trauma in piglets, and gunshot in mature swine [43]. These techniques require very little to no investment in additional equipment and can be effectively carried out once producers are adequately trained in the technique and feel proficient to implement this skill on-farm.

We acknowledge several limitations to this study. With no animal health records to reference, our assessment of the prevention and management of sick animals was based solely on the producer’s response to interview questions and was not verifiable. Similarly, we did not observe euthanasia activities on any of the enrolled farms and relied on producer interview responses to assess that measure. Our interviews with producers and associated farm visits lasted up to 2 h per farm. During this time, the study team aimed to efficiently gather information and make observations. However, it is possible that a longer visit or return visits would have provided a more nuanced level of information. Although the questionnaire was reviewed by in-country study team members and interviews were conducted in Spanish, due to cultural contexts it is possible that question interpretation varied, thus resulting in varied responses.

Across Latin America, as in the Galápagos Islands, the livestock sector is growing, with heightened demands for meat, particularly pork and poultry [26]. Since the World Organization for Animal Health’s first publication on animal welfare principles in 2005, the local emphasis on animal welfare in Latin America, through the implementation of regulations and standards and associated research, has increased [25,26,27]. To date, these efforts have mainly focused on large producers and at industry levels. However, in the Latin American region, smallholder farms are critical in providing food, locally and for the region, in a sustainable way. The evolving attitudes and expectations of local consumers and external international buyers might also influence local practices [47,48]. Looking forward, strategies to enhance animal health and welfare at smallholder farms in Latin America and around the globe should be considered.

## 5. Conclusions

The results of our study demonstrate some of the challenges and opportunities for smallholder farms in remote locations to protect animal health and welfare. Interventions in the realms of animal health management and timely euthanasia are particularly relevant for Floreana Island, and future work should promote knowledge transfer and capacity building in these realms. In accordance with the United Nations Sustainable Development Goals and using a One Health approach, efforts to positively impact smallholder farm livelihoods in Galápagos will sustainably support the interconnected realms of animal health and welfare, wildlife and environmental health, and food safety and security in this unique ecosystem.

## Figures and Tables

**Table 1:** Characteristics of eight production animal farms on Floreana Island, Galápagos Islands—4–5 July 2022.

Farm Characteristics	Farms N (%)
Number of persons who work on the farm	
1	2 (25)
2 or 3	6 (75)
Production animal species on the farm ^1^	
Poultry	8 (100)
Swine	6 (75)
Cattle	5 (62.5)
Multi-species farm ^2^	7 (87.5)
Animal inventory on-farm	
Poultry	
30–50	3 (37.5)
51–80	3 (37.5)
81–300	2 (25)
Swine	
0	2 (25)
1–20	4 (50)
21–80	2 (25)
Cattle	
0	3 (37.5)
1–20	2 (25)
21–67	3 (37.5)

^1^ Multiple production animal species could be selected. ^2^ Two or more production animal species on the farm.

**Table 2:** Assessment of basic animal welfare measures of eight production animal farms on Floreana Island, Galápagos Islands-4–5 July 2022.

Basic Animal Welfare Measures	Farms N (%)
Animal Health Management Common livestock clinical signs or conditions observed by farmers ^1^	
Diarrhea	2 (25)
Respiratory signs	2 (25)
Dystocia and/or Retained Placenta	2 (25)
Lameness or inability to stand	2 (25)
Poor body condition	2 (25)
Parasitic infection ^2^	8 (100)
Timely Euthanasia Recognized five key indicators ^3^ of livestock suffering	
0 to 1 of the five indicators was recognized ^4^	6 (75)
2 to 3 of the five indicators were recognized	2 (25)

^1^ Clinical signs or conditions were [25] included in this table if reported by two or more farms. Producers could report more than one common clinical sign and condition. ^2^ Includes ectoparasites and endoparasites. ^3^ Five key indicators (e.g., behaviors and clinical signs) of livestock suffering: unable to stand, poor body condition, unable to access feed and water, severe injury, and unresponsive to treatment. ^4^ The recognized key indicator for each farm in this group varied.

## Data Availability

The data presented in this study are available on request from the corresponding authors. The data are not publicly available to maintain producer privacy.

## Supplementary Materials

The following supporting information can be downloaded at: https://www.mdpi.com/article/10.3390/ani13040686/s1, S1: Floreana Island producer interview and farm observation data collection tool (English and Spanish versions).
