# Supplementary material for: Assessing Livestock Production Practices on Small-Scale Multi-Species Farms Located on Floreana Island, Galápagos Islands
Source: Animals (Basel). 2023 Feb 16;13(4):686. doi: 10.3390/ani13040686 (PMC9952330; doi:10.3390/ani13040686)
Supplement: Supplementary file 1 [file animals-13-00686-s001.zip › animals-2150141-supplementary.pdf]

<u>Encuesta Sobre La Salud de Los Animales y Gestión Agrícolas - Isla Floreana</u>

<u>Introducción</u>
Veterinario, por favor, lea lo siguiente al agricultor:

- *"Se le invita a participar en un estudio de investigación sobre la, Resistencia a los Antibióticos y Salud Animal en Floreana. Este estudio está siendo realizado por investigadores de la Universidad de NC State en los Estados Unidos. Usted fue seleccionado para participar en este estudio porque tiene una finca con animales en la isla de Floreana. No se trata de una inspección de su finca.*

- *El propósito del estudio es el de obtener un mejor entendimiento de la presencia de resistencia antmicrobiana en las fincas, la vida silvestre, y en aguas residuales humanas en Floreana y determinar la posible relación entre a la resistencia antimicrobiana y la salud animal en Floreana.* Si acepta participar en este estudio, nosotros (el equipo de investigación) le haremos preguntas sobre las prácticas de manejo de la finca y observaremos a sus animales. También recogeremos material fecal animal del suelo y lo analizaremos para determinar si contiene cierto tipo de bacterias (Escherichia coli) que producen enzimas que descomponen los antibióticos de uso común.

- *Es posible que usted no se beneficie directamente de esta investigación; sin embargo, esperamos que su participación en el estudio pueda proporcionar más información sobre la presencia de la resistencia antimicrobiana a través los sectores animales, humanos y del medioambiental en Floreana.*

    - *Con el major esfuerzo, la información que obtenemos para este estudio permanecerá confidencial. No recogeremos su nombre ni el de la finca durante este estudio. Para minimizar cualquier riesgo de la violación de la confidencialidad, eliminaremos cualquier información que pueda identificar la finca de los datos que vamos a analizar.*
    - *Cuando regresamos a Estados Unidos, guardamos los datos originales en papel en una oficina cerrada con llave. Guardamos los archivos de datos electrónicos en una base de datos segura y protegida por contraseña. Todos los datos, las muestras fecales y los materiales relacionados se destruirán al final del estudio de investigación."*

- <u>*Su participación en este estudio es completamente voluntaria y puede dejar de participar en cualquier momento. Puede pedirnos que omitamos las preguntas si hay alguna que no desee responder. Puede hacernos preguntas sobre este proyecto de investigación en cualquier momento.*</u>

- <u>*¿Tiene alguna pregunta sobre el estudio en este momento?"*</u>
  Veterinario, responda a cualquier pregunta.

- <u>*"¿Desea participar en el estudio?"*</u>
  Veterinario, si el agricultor decide participar en este estudio de investigación, pase a la página siguiente.

Información preliminar

1. Número de finca sin identificación ____

2. Fecha y hora de recopilación: fecha ____________________________; hora ___________________________

3. Iniciales del veterinario o estudiante que recopila estos datos: ________________________

4. Tipo de finca (especies de producción): (*Marque todas que se aplican*)

___ Aves

___ Cerdos

___ Bovinos

___ Otro: por favor, describa ____
____
____

5. Número de empleados actuales en la finca: _____________________ (*Indique el número de empleados*)

6. Capacidad de la finca según los productores y/o los empleados (*Indique el número de animales a capacidad*)

Aves: _______________

Cerdos: _____________

Bovinos: ____________

Otro: ______________

Por favor, describa "otro": ____
____

Salud Animal y Evaluación de Bienestar Animal (5 Competencias):

Veterinario, por favor lea la siguiente frase al productor: *"Voy a hacerle algunas preguntas sobre la finca y el ganado*."

Competencia 1: El alimento y las las fuentes de agua de bebida

7. ¿Cuáles son las principales fuentes de agua de bebida para el ganado en la finca? (*Marque todas que se aplican*)

___ Agua de pozo

___ Agua municipal

___ Geomembranas y agua de lluvia recogida en recipientes o tanques en las casas

___ Otra fuente: *por favor, describa* ____
____
____

8a. Si las fuentes principales no están disponibles, ¿hay fuentes secundarias de agua de bebida para el ganado en la finca?

___ Sí – *vaya a la pregunta 8b*

___ No – *vaya a la pregunta 9*

___ No sabe: *por favor, describa* ____
____

*Espacio para comentarios adicionales:*____
____

8b. ¿Cuáles son las fuentes secundarias de agua de bebida para el ganado en la finca? (*Marque todas que se aplican*)

___ Agua de pozo

___ Agua municipal

___ Geomembranas y agua de lluvia recogida en recipientes o tanques en las casas

___ Otra fuente: *por favor, describa* ____
____
____

9. ¿Cuáles son las principales fuentes de alimento para el ganado en la finca? (*Marque todas que se aplican)*

___ Interno / de esta finca

___ Molinos

___ Alimentación comercial

___ Otra fuente: *por favor, describa* ____
____
____

10. Aproximadamente, ¿cuántos días de alimento para el ganado hay en la finca en este momento?

_________ (*Indique el número de días*)

Espacio para comentarios adicionales:

____

____

____

Competencia 2: La protección contra el clima extremo

11. Cuando todo el ganado necesita un lugar de resguardo al mismo tiempo (por ejemplo, si hay condiciones climáticas adversas como lluvia, viento, o calor), ¿hay suficiente alojamiento para que todos los animales estén protegidos?

___ Sí

___ No

___ No sabe: *por favor, describa* ____
____
____

Espacio para comentarios adicionales:
____
____
____

Competencia 3: Animales enfermos y tratamiento

12. ¿Qué signos o condiciones muestra el ganado en la finca cuando está enfermo? (*Marque todos que se aplican*)

___ Diarrea

___ Fiebre

___ Signos respiratorios / dificultad respiratoria / tos / descarga nasal

___ Mastitis

___ Distocicos y retenciones placentarias

___ Infección de la piel

___ Cojera

___ Incapaz de ponerse de pie

___ Mala condición corporal / desnutrición

___ Otra razón no listada aquí: *por favor describa* ____
____
____

___ No sabe: *por favor, describa* ____
____
____

13. En los últimos 12 meses, ¿ha estado un veterinario en la finca por cualquier razón? (*Elija solo una opción*)

___ Sí
___ No

___ No sabe: *por favor, describa* ____
____
____

14a. ¿Hay registros de salud animal en la finca? (*Elija solo una opción*)

***Veterinario**: Si el productor no sabe que es un registro de salud animal, puede explicar: "Los registros de salud animal son cualquier tipo de documentación escrita del cuidado, enfermedad, síntomas, afecciones, medicamentos, u otros tratamientos administrados que son mantendios por el productor o sus empleados."*

___ Sí – **Veterinario**: *por favor, pedirle para ver los registros*

___ No (*Vaya a la pregunta 15*)

___ No sabe: *por favor, describa* ____
____
____

14b. Según los registros de salud animal, ¿se ha tratado algún ganado en la finca en los últimos 12 meses? (Es decir, ¿hay entradas en los registros de salud animal que indican la administración de antibióticos al ganado en la finca en los últimos 12 meses?) (*Elija solo una opción*)

___ Sí

___ No

___ No sabe: *por favor, describa* ____
____
____

14c. ¿Qué fecha es la última entrada en el registro de salud animal?

_____________ (*proporcione la fecha*)

14d. ¿Hay una frecuencia general de tratamientos del ganado se registra en los registros? (por ejemplo: diario, semanal, mensual)

___ Sí: *por favor, describa* ____
____
____

___ No

___ No sabe: *por favor, describa* ____
____
____

15. Cuando un animal de producción está enfermo (véase los ejemplos de la pregunta 12), ¿qué medicamentos están disponibles para uso en la finca? *Describa todos*:

____

____

____

____

16. Según los registros de salud animal y/o el productor, ¿cuáles son los medicamentos más comunes que se utilizan en la finca? *Describa todos:*

____

____

____

____

17. ¿Por qué vía se administraron los medicamentos a los animales de producción en la finca (por ejemplo: oral - añadido al agua o alimento, tópica, inyección intramuscular, inyección subcutánea, otra vía)? *Describa para cada uno de los medicamentos más comunes de pregunta 16:*

____

____

____

____

18. ¿Cómo se almacenan los medicamentos para el ganado? *Describa para cada uno de los medicamentos más comunes de pregunta 16:*

____

____

____

____

19. Específicamente para los medicamentos que son antibióticos, ¿cómo decide el productor dejar de administrar un antibiótico a los animales de producción? (*Marque todos que se aplican*)

___ Basado en el tiempo de tratamiento recomendado por el fabricante/en la etiqueta

___ Basado en las recomendaciones de otro productor, veterinario, o técnico veterinario/ganadero

___ Cuando los animales están mejores (mejora o resolución de los signos y síntomas)

___ Cuando se acaban los antibióticos

___ Cuando cambia la producción animal (por ejemplo, disminución o aumento de la producción de leche o huevos)

___ Cuando los animales no están mejorando

___ Otra razón no listada aquí: *por favor, describa* ____
____
____

___ No sabe: *por favor describa* ____
____
____

Competencia 4: Los animales con comportamientos anormales

20. ¿Qué comportamientos anormales muestran el ganado en esta finca? (*Marque todos que se aplican*)

___ Morder

___ Esconderse

___ Caminar de un lado para otro/caminar ansiosamente

___ Otros signos o síntomas no listado: *por favor, describa.*

He aquí algunos ejemplos:

Aves: arrancarse plumas, canibalismo, amontonamiento
Cerdos masticación simulada, agresión
Bovinos: movimientos de la lengua

____
____
____

__ No sabe: *por favor, describa* ____
____
____

21. ¿Se identifican animales con comportamientos anormales en esta finca? (*Elija solo una opción*)

___ Sí

___ No

___ No sabe: *por favor, describa* ____
____
____

Competencia 5: Eutanasia

22. ¿Qué signos o síntomas muestra el ganado cuando sufren? (*Marque todos que se aplican*)

___ Incapaz de ponerse de pie / debilidad

___ Mala condición corporal / desnutrición

___ Incapaz de acceder alimento y/o agua

___ Lesiones graves

___ No responde al tratamiento

___ Otros signos o síntomas no listados: *por favor, describa* ____________________________________
____
____

___ No sabe: *por favor, describa* ____
____
____

23. ¿Se usa la eutanasia para los animales de producción que sufren?

___ Sí

___ No

___ No sabe: *por favor, describa* ____
____
____

24_ Cuando se aplica la eutanasia a los animales de esta finca, ¿qué método (o métodos) se utilizan para aplicarla? *por favor, describa*
____
____
____
____

Otras Observaciones en la Finca Por Veterinario

25. Además del ganado listado en la pregunta 4, los animales observados en la propiedad incluyen uno o más de las siguientes opciones (*Marque todas que se aplican*):

___ Perros

___ Gatos

___ Roedores

___ Aves silvestres

___ Otra vida silvestre: *por favor, describa* ____
____
____

___ Otros animales: *por favor describa* ____
____
____

26. Se observan uno o más roedores en la propiedad. (*Elija solo una opción*)

___ Sí

___ No

___ No sabe: *por favor, describa* ____
____
____

27. Se observan uno o más productos para el control de los roedores en la propiedad. (*Elija solo una opción*)

___ Sí

___ No

___ No sabe: *por favor, describa* ____
____
____

28a. ¿Cómo se alojan actualmente el ganado en la finca? (*Marque todos que se aplican)*

___ Libre sin cercado (si esta es la única selección, *vaya a la pregunta 29*)

___ Libre con cercado (si esta es la única selección, *vaya a la pregunta 29*)

___ Instalaciones cerrados

___ Otro: *por favor, describa* ____
____
____

28b. ¿Cuántos corrales o instalaciones para los animales de producción hay en la finca actualmente?

___________ (*entrada numérica*)

28c. ¿Son estos corrales o instalaciones a prueba de aves silvestres u otros animales?

___ Sí

___ No

___ No sabe: *por favor, describa* ____
____
____

29. ¿Cómo describiría mejor el tipo o los tipos de refugio para el ganado que hay en esta finca?

___ Si se marca esto, no se debe marcar ninguna otra opción

Marque todas que se aplican:

___ Natural

___ Artificial

____No sabe: *por favor, describa* ____
____
____

30. ¿En qué tipo de piso se aloja el ganado actualmente? (*Marque todas que se aplican*)

___ Tierra

___ Concreto

___ Pasto

___ Grava

___ Otro: *por favor, describa* ____
____
____

31. Hay pediluvios o tinas desinfectantes para zapatos en las entradas de la finca o los corrales/instalaciones de los animales. (*Elija solo una opción*)

___ Sí: *por favor, describa* ____
____
____

___ No

___ No sabe: *por favor, describa* ____
____
____

Animal Health and Farm Management Survey – Floreana Island

Introduction

Veterinarian, please read the following to the farmer:

- *"You are being invited to participate in a research study,* Antibiotic Resistance and Animal Health on Floreana Island*. This study is being done by researchers from North Carolina State University in the United States. You were selected to participate in this research study because you have a farm with animals on Floreana Island. This is not an inspection of your farm.*

- *The purpose of this research study is to better understand the presence of antibiotic resistance on farms, in wildlife, and in human sewage on Floreana and the possible relationship between antibiotic resistance and animal health on Floreana. If you agree to take part in this study, we (the research team) will ask you some questions about management practices on your farm, and we will observe your animals. We will also collect animal fecal material from the ground and test it to determine if is contains a certain type of bacteria (*Escherichia coli*) that produces enzymes that break down commonly used antibiotics.*

- *You may not directly benefit from this research; however, we hope that your participation in the study will provide more information about the presence of antibiotic resistance across animal, humans, and environmental sectors on Floreana.*

    - *To the best of our ability, the information that we collect for this research study will remain confidential. We will not collect your name or the name of the farm during this study. To minimize any risks to breach of confidentiality we will remove any identifying information about the farm from the data that we analyze.*
    - *When we return to the United States, we will keep the original paper data sheets in a locked office. We will store the electronic data files in a secure, password-protected database. All data, fecal samples, and related materials will be destroyed at the end of the research study.*

- *Your participation in this study is completely voluntary and you can withdraw at any time. You can ask us to skip question(s) if there are any questions that you do not want to answer. You can ask us questions about this research project at any time.*

- *Do you have any questions about the study at this time?"*
  Veterinarian, answer any questions.

- *"Would you like to participate in the study?"*
  Veterinarian, if the farmer elects to participate in this research study, proceed to the following page.

Background Information

1_De-identified farm number: ____ (*free text*)

2_Date and time of data collection: *date*__________________; *time*____________________ (*formatted*)

3_Initials of veterinarian (or veterinary student) collecting these data: ___________________ (*free text*)

4_Farm type (production species): (*Check all that apply*)

__Poultry

__Swine

__Cattle

__Other: please describe (*free text*) _____

5_Number of persons currently employed by the farm: ___________________ (*formatted numerical*)

6_Farm capacity according to the producer/farm employee(s) (*formatted numerical*)

Poultry: __________

Swine: ___________

Cattle: ___________

Other: ____________

Please describe "other": ____
____

Animal Health & Welfare Assessment (5 Competencies)

Veterinarian, please read the following to the producer: *"I am going to ask some questions about the farm and the livestock."*

Competency 1: Feed & Drinking Water Sources

7_What is/are the main source(s) of livestock drinking water on the farm? (*Check all that apply*)

__Well

__Municipal water

__Geomembranes and rainwater collected in containers or tanks in the houses

__Other source: please describe (*free text*)
____
____
____
____

8a_Is there one (or more) back-up source(s) of livestock drinking water on the farm if the main source(s) become(s) unavailable?

__Yes – *Go to 8b*

__No – *Go to 9*

__Not sure please describe (*free text*)
____
____
__

Additional free text space: ____
____
____
____

8b_What is (are) the back-up source(s) of livestock drinking water on the farm? (*Check all that apply*)

__Well

__Municipal water

__Geomembranes and rainwater collected in containers or tanks in the houses

__Other source: please describe (*free text*)
____
____
____
____

9_What is/are the main source(s) of livestock feed on the farm? (*Check all that apply*)

__Internal / from this farm

__Mill

__Commercial product

__Other source: please describe (*free text*)
____
____
____
____

10_Today, approximately how many days-worth of livestock feed is on the farm?

_________ (*Numerical days)*

Additional free text space: ____
____
____
____

Competency 2: Protection from Extreme Weather

11_When all the livestock need shelter at the same time (for example, if there are adverse weather conditions like excessive rain, wind, or heat) is there enough housing for all the animals to be sheltered?

__Yes

__No

__Not sure: please describe (*free text*)
____
____

Additional free text space: ____

Competency 3: Sick Animals and Treatment

12_What signs or conditions do the livestock on the farm show when they are sick? (*Check all that apply*)

__Diarrhea

__Fever

__Respiratory signs / trouble breathing / coughing / nasal discharge

__Mastitis

__Dystocia and/or retained placenta

__Skin infection

__Lameness

__Unable to stand

__Poor body condition

__Another reason not listed here: please describe *free text*)
____
____
____
____

__Not sure: please describe *free text*)
____
____
____
____

13_During the past 12 months, has a veterinarian been on the farm for any reason? (*Choose only one*)

__Yes

__No

__Not sure: please describe (*free text*)
____
____
____
____

14a_ Are animal health records maintained on the farm? (*Choose only one*)

***Veterinarian**: If the producer is not sure what an animal health record is, you can prompt them: "Animal health records are any type of written documentation of animal care, disease, clinical signs, conditions, medications, or other treatments administered that are maintained by the producer or their employee(s)."*

__Yes – **Veterinarian**: *please ask to see the animal health records*

__No (*Go to Q15*)

__Not sure: please describe (*free text*)

____

14b_ According to the animal health records, have any livestock on the farm been treated in the past 12 months? (i.e., Are there any entries in the animal health records within the past 12 months indicating that livestock have been treated?) (*Choose only one*)

__Yes

__No

__Not sure: please describe (*free text*)

____

14c_What is the date of the last entry in the animal health record? ____________________________ (*free text*)

14d_Is there a general frequency or cadence (e.g., daily, weekly, monthly) in the livestock treatments as recorded in the animal health records?

__Yes: please describe (*free text*)

____

__No

__Not sure: please describe (*free text*)

____

____

____

_____

15_When a production animal on the farm is sick (*see Q12 for examples*), what medication(s) are available for use on the farm? *Describe/list all:*

____
____
____
____
__
____
____
____
____
__

16_What is/are the most common medication(s) that are used on the farm (according to the animal health records and/or the producer)? *Describe/list all:*

____
____
____
____
__
____
____
____
____
__

17_How are the most common medication(s) (Q16) administered to the production animals on the farm? (e.g., oral in feed/water, topical, intramuscular injection, subcutaneous injection, other route) *Describe for each of the common medication(s) from Q16*:

____
____
____
____
__
____
____
____
____
__

18_How are the common medication(s) (Q16) for the livestock stored? *Describe for each of the common medication(s) from Q16:*

____
____
____

19_Specifically, for medications identified as antibiotics:

How does the producer decide to stop administering the antibiotic to the production animal(s)? (*Select all the apply*)

__Based on treatment time recommended by the manufacturer / on the label

__Based on advice from another farmer, veterinarian, veterinary/livestock technician

__Animal(s) is(are) better (improved or resolved signs or symptoms)

__When the medicine(s) is/are gone (when we run out of antibiotic(s))

__When animal production changes (e.g., decreased or increased milk or egg production)

__When the medicine(s) is/are not making the animal(s) better

__For another reason, not listed: please describe (*free text*)
____
____
____
____

__Not sure: please describe (*free text*)
____
____
____
____

Competency 4: Animals with Abnormal Behaviors

20_What abnormal behaviors do the livestock show on this farm? (*Check all that apply*.)

__Bites

__Hides

__Paces/walks anxiously

__Other signs or symptoms not listed:

He aquí algunos ejemplos:
Poultry: feather pecking, cannibalism, piling
Swine sham chewing, aggression
Cattle: tongue rolling

please describe (*free text*)
____
____
____

__Not sure: please describe (*free text*) ____
____
____

21_Are animals with abnormal behaviors on this farm identified? (*Check only one*.)

__Yes

__No

__Not sure: please describe (*free text*)
____
____
____
____

Competency 5: Euthanasia

22_What signs or symptoms do the livestock show when they are suffering on this farm? (*Check all that apply.*)

__Unable to stand / weak

__Poor body condition

__Unable to access food and/or water

__Severe injury

__Not responding to treatments

__Other signs or symptoms not listed: please describe (*free text*) ____

__Not sure: please describe (*free text*) ____

23_Are animals that are experiencing suffering on this farm euthanized? (*Check only one*)

__Yes
__No
__Not sure: please describe (*free text*) ____

24_What method (or methods) does the farm use to euthanized animals?

Please describe (*free text*): ____

Other On-farm Observations Made by the Veterinarian

25_ *Other than* the livestock species (indicated in Q4), animals observed on the property include one or more of the following (*Select all that apply*):

__Dogs

__Cats

__Rodents

__Birds (other than chickens)

__Other Wildlife: please describe (*free text*)
____
____
____
____

__Other animal(s): please describe (*free text*)
____
____
____
____

26_ One or more rodent(s) are observed on the property. (*Choose only one*)

__Yes

__No

__Not sure: please describe (*free text*)
____
____
____
____

27_ One or more rodent control device(s) and/or substance(s) are observed on the property. (*Choose only one*)

__Yes: please describe (*free text*)
____
____
____
____

__No

__Not sure: please describe (*free text*)

____

____

____

_____

28a_ How are the livestock on the farm currently housed? (*Check all that apply)*

__Open or free roaming with no fencing (If this is the only selection, skip to Q29)

__Open or free roaming with fencing (If this is the only selection, skip to Q29)

__Closed housing enclosures

__Other: please describe (*free text*)

____

28b_ How many animal enclosures or buildings are currently on the farm?

(Numerical free text) ________________

28c_ Are these enclosures/buildings bird-proof or other animal-proof?

__Yes

__No

__Not sure: please describe (*free text*)

____

29_How would you characterize the type(s) of livestock shelter that is/are on the farm?

__ This question does not apply because there is no livestock housing or shelter on the farm. (If this is checked, no other option should be checked)

Or, check all that apply:

__ Natural

__ Artificial

__ Not sure: please describe (*free text*)

____

Additional free text space: ____
____
____
____

30_ What type of flooring are the livestock on the farm currently housed on? (*Check all that apply*)

__Soil

__Concrete

__Grass

__Gravel

__Other: please describe (*free text)*
____
____
____
____

31_ Foot baths or boot dips are present at entrances to the farm or at entrances to animal enclosure(s). (*Choose only one*)

__Yes please describe (*free text*)
____
____
__
____
__

__No

__Not sure: please describe (*free text*) ____
____
____
____
